# Bilateral Synchronous Testicular Cancer with Discordant Histopathology: A Case Report

**DOI:** 10.1055/s-0044-1786512

**Published:** 2024-04-29

**Authors:** Mehmet Özen, İrem Yazıcıoğlu, Mustafa Koray Kırdağ, Mustafa Aydın

**Affiliations:** 1 Department of Urology, Samsun Education and Research Hospital, Samsun, Turkey; 2 Department of Pathology, Samsun Education and Research Hospital, Samsun, Turkey

**Keywords:** testicular tumor, radical orchiectomy, embryonal carcinoma, seminoma

## Abstract

Bilateral testicular tumors account for 1 to 5% of all testicular tumors. Most bilateral tumors are observed metachronously. Synchronous tumors usually present with the similar histological pattern. Bilateral synchronous testicular tumors with discordant pathology are extremely rare. Only 56 cases have been documented since Bidard first described synchronous testicular tumors with discordant pathology in 1853. To our best knowledge, this study will be the 57th case in the literature.

## Introduction

Testicular tumors are the most common malignant tumors in men aged between 15 and 45, accounting for 1% of tumors in male patients and 5% of urological cancers. 1 Bilateral germ cell testicular cancer occurs in 1 to 5% of testicular tumors. 2 Most cases are metachronous and approximately 10% being synchronous. Synchronous tumors often show similar histological features and classifications with frequent involvement by seminoma. Rarely, synchronous tumors show two different tumors. 3

The purpose of this study is to present a case of synchronous bilateral germ cell testicular tumor that has different histological features.

## Case Presentation

A 32-year-old male with cerebral palsy presented with painless swelling in both testes that occurred within a month. Physical examination revealed painless scrotal swelling and deformities in both lower extremities. The patient's medical history was unclear, and mental retardation was not observed.

Tumor markers showed a normal α-fetoprotein level of 1.49 ng/mL (normal range: <7.0 ng/mL), elevated β-human chorionic gonadotropin level of 255 mIU/mL (normal range: <5.0 mIU/mL), and elevated lactate dehydrogenase level of 815 IU/mL (normal range: 91–180 IU/mL). On scrotal ultrasonography (USG), there was an accumulation of fluid around both testicles. In addition, USG revealed hypoechoic multicystic lesions in both testicles, but it was not possible to differentiate between tumors and testicular abscesses. Magnetic resonance imaging was not possible due to deformity of the extremity. Abdominal and thoracic computed tomography scans were performed, revealing a 4-cm mass in the left paraaortic region of the abdomen and 8.5 and 5.5-cm masses in the thorax. Subsequently, bilateral radical orchiectomy was performed. Sperm banking was offered; however, the patient declined.

**Fig. 1:**
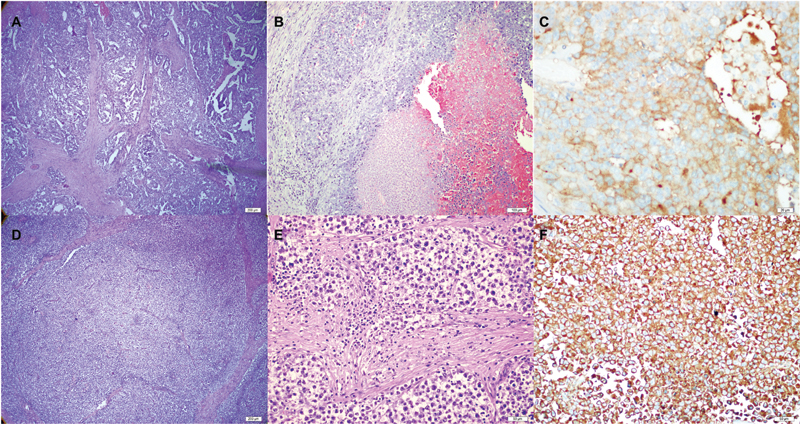
( **A** ) Right-sided embryonal carcinoma, glandular and papillary pattern (x40, hematoxylin and eosin). ( **B** ) Right-sided embryonal carcinoma, area with hemorrhagic necrosis (x100, hematoxylin and eosin). ( **C** ) Right-sided immunohistochemical stain showing CD30 expression on a section of the embryonal carcinoma (x40). ( **D** ) Left-sided seminoma, tumor cells containing fibrous septa (x40, hematoxylin and eosin). ( **E** ) Left-sided seminoma, cells with prominent nucleoli and transparent cytoplasm (x200, hematoxylin and eosin). ( **F** ) Left-sided immunohistochemical stain showing PLAP expression of the seminoma (x20) PLAP, placental alkaline phosphatase.

Histopathological evaluation of the right testicle revealed embryonal carcinoma with invasion of the tunica albuginea. No lymphovascular invasion or invasion of the tunica albuginea was observed. The tumor on the right side measured 8.5 × 4.5 cm. The tumor on the left side measured 9.5 × 7.5 cm and was revealed to be seminoma with lymphovascular invasion and invasion of the rete testis ( Fig. 1 ). The levels of tumor markers remained elevated 1 month after surgery. After being referred to the oncology department, the patient underwent chemotherapy with bleomycin, etoposide, and cisplatin.

## Discussion

Testicular cancer is the most common solid tumor in men under the age of 40. 2 In 2022, an estimated 9,910 patients were diagnosed with testicular tumors in the United States. 4 The incidence of bilateral germ cell testicular tumors ranges from 1 to 5%. 2 Testicular tumors are classified as either synchronous or metachronous. Synchronous tumors represent tumors monitored at the counter test during or within the first 2 months of diagnosis. 5 Approximately 80 to 85% of the cases are reported as metachronous, while remaining cases are synchronous. This means that synchronous bilateral germ cell tumors account for less than 0.5% of all testicular tumors.

The first case of bilateral testicular tumor was presented by Bidard in 1853. Since then, several studies have been published on bilateral testicular tumors. Holzbeierlein et al reported one of the widest series of testicular tumors, monitoring only 10 patients with 3,984 tumors for synchronous bilateral testicular tumors. 6 In a study by Che et al in which 2,431 patients with testicular tumors were evaluated, they found bilateral testicular tumors in 24 patients and showed that only 4 of them were synchronous. 7 Akdogan et al have detected bilateral tumors in 30 out of 987 patients, with only 6 of them being synchronous. 8

Most synchronous bilateral germ cell tumors present with seminoma histological pattern in both testes. Dieckmann et al demonstrated that 80% of synchronous bilateral germ cell testicular tumors were seminomas. 9 Although rare, different histological types of synchronous tumors have been reported. 10 The first case of bilateral testicular tumors with different histological types was reported by Coleman et al in 1954. 6 In 2003, Coli et al reported the 43rd case of bilateral testicular tumors with different histological types. 11

In 2015, the 49th case report was published by Anastasiou et al. 6 Since then, we found only seven cases by searching the PubMed's and Google Scholar's databases. 2 3 10 12 13 14 15 Therefore, our case is the 57th synchronous bilateral testicular tumor case with different histology to have been reported up to date, according to PubMed's and Google Scholar's databases.

## Conclusion

Synchronous bilateral germ cell tumors of the testicles are a rare occurrence. Although only a small number of documented cases have been reported with a different histology, the majority of cases present with the same histologic type, typically seminoma. Despite their rarity, bilateral testicular tumors should be approached in the same way as unilateral tumors, with radical orchiectomy being the standard of care.
